# The effects of the COVID-19 pandemic on drug and poison–related deaths in Gold Coast, Australia

**DOI:** 10.1007/s12024-022-00555-5

**Published:** 2022-11-18

**Authors:** Isabella Thompson, Alex Olumbe, Rexson Tse, Melissa Thompson

**Affiliations:** 1 Faculty of Health Sciences and Medicine, Bond University, Robina, QLD Australia; 2 Forensic and Scientific Services, Health Support Queensland, Brisbane, QLD Australia; 3 School of Medicine and Dentistry, Griffith University, Southport, QLD Australia; 4 Department of Molecular Medicine and Pathology, University of Auckland, Auckland, New Zealand

**Keywords:** Postmortem, Drugs, Alcohol, Poison, Pandemic, COVID-19

## Abstract

**Introduction:**

COVID-19 is postulated to impact drug- and poison-related deaths. America has reported an increased in drug-related deaths, whereas Australia has reported a decline. Regional studies are scant and may not mirror national data. Characterising drug and poison–related deaths during COVID-19 at a regional level would inform local interventions and policies on the current and future pandemics.

**Methods:**

A 4-year retrospective study from January 1, 2018, to December 31, 2019 (pre-COVID-19 pandemic) and from January 1, 2020, to December 31, 2021 (COVID-19 pandemic) of all drug and poison–related deaths admitted to the Gold Coast University Hospital under Coronial investigation.

**Results:**

Drug and poison–related deaths increased in both the proportion and absolute numbers before and during the COVID-19 pandemic. There was no statistical difference in age, sex, location of death, manner of death and classification of drugs and poison implicated.

**Conclusions:**

Although there is an increase in drug and poison–related deaths, the overall demographic and pattern have not changed. Further studies to account for the variation may enable implementation of targeted public health interventions to address the burden of related deaths in regional settings in the context of future pandemics.

## Introduction

The 2019 novel coronavirus causes severe acute respiratory syndrome (COVID-19) [1]. With the global outbreak of COVID-19, it was declared a pandemic in March 2020. Governmental authorities worldwide implemented a variety of public health measures to reduce the spread of COVID-19. These measures targeted the entire spectrum of the public health system at individual, community, national and international levels [2]. These measures included vaccination, personal protective equipment (such as face mask/coverings and gloves), self-isolation, social distancing, closure of non-essential services, working from home, limiting public gatherings, travel restrictions and closing of national and international borders. However, these measures are not uniformly implemented and adhered as variation at international, national, regional and personal levels is reported in literature and in the media [3–6].

Drug and poison–related deaths are a major public health concern worldwide, with some countries describing it as an epidemic/crisis, especially for drugs such as opioids [7–10]. Drugs involved include illicit, prescribed or over-the-counter medications and alcohol. Types of poison used commonly include household chemicals or toxic substances or fumes. The choice of drug and poison use depends on availability to the individual. With the COVID-19 pandemic, it was expected that there would be a change in drug and poison use dynamics reflecting a change in availability and accessibility [11]. This is further complicated by changes in access to medical care while managing the COVID-19 pandemic. As such, America have reported an increase in deaths from drug overdose during the pandemic, but this was not mirrored in Australian data in preliminary reports [11, 12]. This highlights the variable impact of the pandemic on drug and poison–related deaths at an international level.

With the variability of the impact of COVID-19 public health measures and pre-existing drug and poison use dynamics, it is foreseeable that regional data may differ from national data. However, regional data on drug and poison–related deaths during the COVID-19 pandemic is scant. It is unclear whether the change in these types of deaths observed at a national level during COVID-19 is mirrored at a regional level. Characterising drug and poison–related deaths during COVID-19 at a regional level would inform local interventions and policies on the current and future pandemics. This retrospective study aims to characterise drug and poison–related deaths before and during the COVID-19 pandemic in regional Australia (Gold Coast, Queensland, Australia).

## Materials and methods

A 4-year retrospective study from January 1, 2018, to December 31, 2019 (pre-COVID-19 pandemic) and from January 1, 2020, to December 31, 2021 (COVID-19 pandemic) on all drug and poison–related deaths admitted to the Gold Coast University Hospital (GCUH) under Coronial investigation was performed. This department is responsible for post-mortem examination of all decedents referred to the coroner from the Gold Coast and surrounding areas and is considered a regional department.

Postmortem reports were reviewed for each case and data retrieved (manual chart review). Using the postmortem date, the cases were divided into two groups, being pre-COVID-19 pandemic (pre-COVID, January 1, 2018, to December 31, 2019), and during COVID-19 pandemic (COVID, January 1, 2020, to December 31, 2021). Age, sex, socioeconomic demographics, cause of death and results from the toxicological report were recorded. The location (home, community, hospital) and manner of death was also recorded (deliberate or accidental).

In the toxicological report, the implicated drugs causing death were categorised as alcohol, illicit (stimulants, opioids, hallucinogens), prescription (including over-the counter), others and mixed (illicit, prescription and/or alcohol). Others included any form of toxicity such as toxic substances and fumes.

### Statistical analysis

Statistical analysis was performed using R 3.6.3 Open source and a *p*-value of < 0.05 was considered significant. Continuous variables were presented as mean, median, minimum, maximum and standard deviation (s.d). Discrete variables were represented as counts. Student *t*-test was used to compare continuous variables (Kolmogorov–Smirnov test was performed to test for normality), and chi-square test was used to compare categorical variables between the two groups (pre-COVID and COVID).

### Ethics

This study was approved by the Support Health Queensland, Forensic Scientific Services Human Ethics Committee (FSS-HEC 21–26).

## Results

A total of 202 cases were identified during the two periods, with a mean age of 47.03 and an overall predominance of male (M:F: 133:79). There were 118 cases classified as deliberate and 146 (majority) cases were found dead at home.

The drugs implicated for the death were commonly illicit, prescribed and mixed drug toxicity. The most common illicit drug classifications were stimulants and opioids with lower numbers of hallucinogens and novel psychoactive substances. Common prescribed and over-the-counter drugs were prescription opioids, sedatives, benzodiazepines and psychiatric medications. Other substances (poisonous) included sodium nitrate, glyphosphate, isopropyl alcohol, ethylene glycol, carbon monoxide, butane and nitrogen inhalation and hydrogen sulphide. A summary between pre-COVID and COVID is shown in Table 1.

**Table 1:** Comparison of drugs and poison–related deaths pre-COVID-19 and COVID-19 pandemic in Gold Coast, Queensland, Australia

Time period	Number of cases (Percentage of total case)	Mean age (years)	Sex (M:F)	Location of death			Manner of death		Drug and poison			
				Home	Community	Hospital	Accidental	Deliberate	Illicit	Prescription	Mixed	Others
Pre-COVID-19 pandemic	83 (9.5%)	47.04	52:31	54	15	14	32	51	36	17	22	5
COVID-19 pandemic	129 (13.7%)	47.02	81:48	92	23	8	62	76	24	27	42	14

### Pre-COVID

A total of 83 cases were identified as drug and poison–related deaths in the pre-COVID group which was 9.5% of the total coronial deaths (877 cases) admitted to the GCUH. The mean age was 47.04 (min: 20, max: 92, median: 45 and s.d: 15.0) with a male predominance (M:F: 52:31). Fifty-one cases were classified as deliberate drug and poison–related deaths and 32 cases were classified as accidental. Fifty-four cases died at home, 15 died in community and 14 died in hospital.

In terms of drug classification, cases showed an overall predominance of prescription and mixed drug and poison–related deaths. Thirty-six cases were attributed to prescription medication, 22 from mixed drugs, 17 from illicit drugs, 5 cases from other substances (3 cases from carbon monoxide, and one case each from isopropyl alcohol and ethylene glycol) and 3 cases from alcohol.

The most detected illicit drugs were methamphetamine (19 cases) and heroin (9 cases). The most detected classes of prescription medications were benzodiazepine (53 cases) and antidepressants (12 cases).

### COVID

A total of 129 cases were identified as drug and poison–related deaths in the COVID group which was 13.7% of the total coronial deaths (942) admitted to the GCUH. Similarly, the overall mean age was 47.02 (min:16, max: 93, median: 46 and s.d: 16.6) with a male predominance (M:F: 81:48). Sixty-seven cases were classified as deliberate drug-related death and 62 cases were classified as accidental. Ninety-two cases died at home, 23 died in community and 8 died in hospital.

In terms of drug classification, there was an overall predominance of prescription and mixed drug and poison–related deaths. Twenty-seven cases were attributed to prescription medication, 42 from mixed drugs, 24 from illicit drugs, 14 cases from other substances (6 from carbon monoxide, 3 from sodium nitrate, 2 from hydrogen sulphide, one case each from butane and nitrogen inhalation and glyphosate) and 3 cases from alcohol.

The most detected illicit drugs were methamphetamine (19 cases) and heroin (11 cases). The most detected classes of prescription medications were benzodiazepine (74 cases) and antidepressants (13 cases).

### Statistical comparison between pre-COVID and COVID

The overall number and proportion of drug and poison–related deaths was higher in the COVID group (129 cases, 13.7%) compared to the pre-COVID group (83 cases, 9.5%).

The age and sex between the two groups were not significantly different (age: *t*-test, *p-*value = 1.00; sex: chi-square, *p*-value = 0.98). Also, there was no significant difference in the manner of death between the two groups (Chi-square, *p*-value = 0.17). The location of death also did not reach (but trended) statistical significance (Chi-square, *p*-value = 0.06).

Comparing the pre-COVID and COVID group, there was no significant difference in the class of drug and poison used (chi-square, *p-*value = 0.47).

## Discussion

This regional study on drug and poison–related deaths before and during COVID-19 pandemic showed an increase in both the proportion and absolute numbers of drug and poison–related deaths during COVID-19. The common detected illicit drugs and class of prescription medications were the same before and during COVID-19 pandemic. Methamphetamine, heroin, benzodiazepine and anti-depressant being the most commonly detected drugs and medication in study periods. Apart from occasional novel poisonous substances detected, there was no statistical difference in age, sex, location of death, manner of death and classification of drugs and poison implicated before and during the COVID-19 pandemic. This suggests that although there is an increase in drug and poison–related deaths, the overall demographic and pattern of drug and poison use have not changed over the two periods studied.

### National data

Our study contrasts with preliminary national data from the Australian Institute of Health and Welfare showing a decrease in total, accidental and deliberate drug-related deaths during the pandemic [12]. It is possible that this divergence may be due to the lower COVID-19 case burden and restrictions in Queensland relative to other states, allowing for greater access to essential services such as medical centres and pharmacies to access prescription and over-the-counter medications and minimal disruptions to gatherings allowing for continued trafficking of illicit substances and use in group settings. Geographical factors may also play a role given the region’s coastal setting, enabling maintenance of illicit supply into Queensland via sea transport, despite the anticipated supply disruptions due to restrictions on international air travel [13]. Another explanation is the commute between Gold Coast and neighbouring state despite inter-state boarder closure. Gold Coast is in proximity with another neighbouring regional city (Tweeds Head, New South Wales) and commute between the two states and city are easy and frequent due to living and working arrangements.

However, local metropolitan jurisdictions have also experienced similar rises in drug-related service use. A study of emergency department presentations to Westmead Hospital, New South Wales related to substance use disorders comparing admission rates from January to July 2020 to the same period in 2019 found a significant rise in admissions from 1.7 to 3.4% [14]. This is in the context of a concerning increase in mental health presentations for the Western Sydney Local Health District, New South Wales, from March to May 2020 to the equivalent period in 2019, despite an overall reduction in emergency department admissions early in the pandemic [14, 15].

There is unfortunately limited availability of local mortality data to draw inter-regional comparisons or compare regional and metropolitan areas at a state-based level to identify whether this trend has been the experience of other regional areas of Australia. However, our study provides evidence that regional drug-related deaths may not mirror national trends during the COVID-19 pandemic.

### International data

Our findings are consistent with international trends in drug-related deaths coinciding with the event of the COVID-19 pandemic. A recent systematic analysis of drug overdose-related deaths in the USA and Canada comparing pre- and post-pandemic death rates found drug-related deaths increased during the pandemic by up to 60% in certain US jurisdictions and by 58% in Canada from the first to second quarter of 2020 [11]. In contrast, data from the European Union showed mixed changes in drug-related deaths [16]. While the total drug-related deaths in 2020 increased in Finland, Czechia reported stable numbers while Italy and Portugal’s preliminary data showed a reduction relative to 2019 [16]. In addition, a US study leveraging mortality records from the Centre for Disease Control & Prevention found that overdose mortality climbed by 34.8% for the period from January to July 2020 compared with the same period in 2019, peaking at 57.7% in May 2020 [17]. A Canadian study utilising surveillance data from the British Columbia Coroners Service found deaths from overdose of illicit substances more than doubled from March to December 2020 relative to 2019 [18]. It has been postulated that macroenvironmental repercussions of the COVID-19 pandemic such as increased social isolation, mental health stressors, unemployment and economic insecurity may underpin the changes demonstrated globally in drug-related mortality [19]. This trend of increasing drug-related mortality is supported by a large cross-sectional study of US emergency department presentations which found significantly elevated median counts for overdoses and suicide attempts between March and October 2020 relative to counts over the same period in 2019 [20].

At a state level, West Virginia, Kentucky and Tennessee experienced the highest per capita drug-related mortality in May 2020 [17]. Rhode Island, which in December 2020 had the highest rate of COVID-19 cases and mortality in the USA, documented similar increases in drug-related deaths in 2020 compared with 2019, with an overall increase in mortality by 28.1% from 29.2 per 100,000 person-years in 2019 to 37.4 per 100,000 person years [19]. This is consistent with findings in Ohio, San Francisco and California [21–23].

In terms of demographics, there was an increased in illicit drug-related deaths in males and people aged 40 and above [18]. Similarly, other states found a predominance of males in overdose deaths during 2020 [22, 23]. This was divergent from findings of the present study and other American regional studies which found no significant difference in the sex distribution of cases before and during COVID-19 [19, 21, 24]. There is a lack of consensus with regard to age with conflicting findings in the literature. A study reported most overdose deaths occurring between 18 and 64 years of age, another found similar trends over monthly intervals in all age groups, with the largest spike occurring in the youngest age group (≤ 24) [22, 23]. In contrast, other studies found a 2.6% increase in the average age of overdose death cases, and another reported no significant difference in the age of drug-related deaths in 2020 compared to 2019 [21, 24]. These studies again highlight the variability between different regions in drug-related deaths during COVID pandemic.

### Limitations

As this study is observational in nature, it is not possible to establish a causal relationship between the COVID-19 pandemic and observed increases in drug-related mortality in the Gold Coast region during the pandemic. It is plausible that other factors may confound this association. As this study focused on a regional area, the results are not readily generalisable to the metropolitan or national context.

We included poisonous substances (as others in this study) but did not further analyse the specific implicated substances due to small number of cases. The results may vary slightly when compared to other drugs only studies in literature.

## Conclusions

This retrospective study of drug and poison–related deaths occurring pre- and post-COVID-19 in Gold Coast, Australia, presents evidence of increased drug and poison mortality following the COVID-19 pandemic. This is divergent from preliminary data at a national level, suggesting a possible variable impact of COVID-19 in regional areas of Australia which has implications for future funding, policy development and enhancement of service delivery. Further research is required to characterise the possible impact of COVID-19 on regional and metropolitan drug and poison–related mortality and the underlying mechanisms accounting for variation. This would enable implementation of targeted public health interventions to address the burden of drug-related deaths in regional settings in the context of future pandemics and other significant global events.

## Key points

1. The effect of COVID-19 pandemic on drug and poison–related is variable at an international level.
2. Regional data on drug and poison–related deaths during the COVID-19 pandemic is scant.
3. Australia reported a decrease in drug and poison–related deaths during COVID-19 pandemic.
4. Contrary to national data, we report an increase in drug and poison–related deaths in Gold Coast, Queensland.
5. Apart from an increase, the overall characteristics of drug and poison–related deaths are unchanged.
